# Genome-Wide Investigation of the CRF Gene Family in Maize and Functional Analysis of ZmCRF9 in Response to Multiple Abiotic Stresses

**DOI:** 10.3390/ijms25147650

**Published:** 2024-07-12

**Authors:** Zhenwei Yan, Jing Hou, Bingying Leng, Guoqi Yao, Changle Ma, Yue Sun, Fajun Zhang, Chunhua Mu, Xia Liu

**Affiliations:** 1 Maize Research Institute, Shandong Academy of Agricultural Sciences, Jinan 250100, China; yanzwplant@sina.com (Z.Y.); lengbingying305@126.com (B.L.); yaoguoqi@saas.ac.cn (G.Y.); muchunhua@saas.ac.cn (C.M.); 2 School of Agriculture, Ludong University, Yantai 264001, China; houjing@m.ldu.edu.cn; 3 College of Life Sciences, Shandong Normal University, Jinan 250300, China; machangle@sdnu.edu.cn; 4 College of Agronomy, Qingdao Agricultural University, Qingdao 266109, China; sunyue3070601@163.com

**Keywords:** maize, cytokinin response factors, genome-wide analysis, expression analysis, abiotic stress

## Abstract

The cytokinin response factors (CRFs) are pivotal players in regulating plant growth, development, and responses to diverse stresses. Despite their significance, comprehensive information on *CRF* genes in the primary food crop, maize, remains scarce. In this study, a genome-wide analysis of *CRF* genes in maize was conducted, resulting in the identification of 12 members. Subsequently, we assessed the chromosomal locations, gene duplication events, evolutionary relationships, conserved motifs, and gene structures of all ZmCRF members. Analysis of *ZmCRF* promoter regions indicated the presence of cis-regulatory elements associated with plant growth regulation, hormone response, and various abiotic stress responses. The expression patterns of maize *CRF* genes, presented in heatmaps, exhibited distinctive patterns of tissue specificity and responsiveness to multiple abiotic stresses. qRT-PCR experiments were conducted on six selected genes and confirmed the involvement of *ZmCRF* genes in the plant’s adaptive responses to diverse environmental challenges. In addition, ZmCRF9 was demonstrated to positively regulate cold and salt tolerance. Ultimately, we explored the putative interaction partners of ZmCRF proteins. In summary, this systematic overview and deep investigation of ZmCRF9 provides a solid foundation for further exploration into how these genes contribute to the complex interplay of plant growth, development, and responses to stress.

## 1. Introduction

The plant phytohormone cytokinin (CK) plays a crucial role in governing plant growth, development, and responses to abiotic stress [1,2,3,4]. The signaling pathway of cytokinin in plants functions through a canonical two-component signaling system [5,6]. Briefly, cytokinin binds to histidine kinase receptors, such as those found in *Arabidopsis thaliana* (AHK receptors). This binding leads to auto-phosphorylation of the receptors, followed by the transfer of the phosphoryl group to Arabidopsis histidine phosphotransfer proteins (AHPs). Subsequently, the phosphoryl group is transferred to type-A and type-B Arabidopisis response regulators (ARRs), thus modulating the expression of downstream target genes.

Within the context of this signaling pathway, cytokinin response factors (CRFs) emerge as a distinct subclade within the APETALA2 (AP2)/ETHYLENE RESPONSIVE FACTOR (ERF) family of transcription factors (TFs), found throughout the plant kingdom [7]. Functioning as a parallel branch to the canonical cytokinin two-component signal transduction pathway, CRF proteins exhibit a conserved AP2 DNA-binding region [8]. Aside from the conserved AP2 DNA-binding region, CRF proteins also feature a phylogenetic group-specific CRF domain comprising approximately 65 amino acids [9]. This CRF domain is responsible for facilitating protein–protein interactions [10]. Moreover, CRF proteins possess a variable C-terminal region [11]. In addition to their potential independent regulation of downstream targets, CRF proteins have the capacity to transfer and amplify the cytokinin signal in collaboration with type-B response regulators [12].

CRFs are ubiquitously involved in plant growth and development and contribute significantly to stress-response networks [7]. For instance, through analysis of loss-of-function mutations, M. Rashotte et al. revealed that AtCRF1/2/5 redundantly control the development of embryos, cotyledons, and leaves [8]. Beyond their involvement in normal plant growth and development, CRF proteins have also been identified to participate in multiple stress responses. In the model plant *Arabidopsis*, exposure to cold stress results in the induction of *AtCRF2* and *AtCRF3*, which in turn enhances tolerance to cold by regulating lateral root initiation and development [13]. AtCRF4, which is significantly induced by cold treatment, was found to contribute to freeze tolerance [14]. In the case of *Tamarix hispida*, ThCRF1 exhibits a response to salt stress, endowing the plant with salt tolerance through the modulation of osmotic potential and augmentation of its capacity to scavenge reactive oxygen species [15]. Furthermore, Wang et al. reported that *Brassica napus* BnaCRF8s specifically govern phosphate homeostasis and root architecture in response to phosphate fluctuation [16]. Additionally, several studies have also highlighted the potential roles of plant CRF proteins in oxidative stress, heat stress, and flooding stress [17,18,19,20].

To date, CRF proteins have only been identified and characterized in a limited number of species, including 12 members (AtCRF1-AtCRF12) in *Arabidopsis thaliana* [8,9], 11 members (SlCRF1-SlCRF11) in tomato [21], 21 members (BrCRF1-BrCRF21) in Chinese cabbage [22], 44 members (BnaCRF1-BnaCRF44) in *Brassica napus* [16], and 26 members (GmCRF1-GmCRF26) in soybean [23]. However, detailed information about *CRF* genes in other species remains elusive, and their biological functions require further exploration.

Maize (*Zea mays* L.), a major global grain and forage crop, is a key resource for food and bioenergy industries [24]. Maize, as the largest grain crop in the world, plays a vital role in sustaining food security globally. Up to 20% of food calories in sub-Saharan Africa, Southeast Asia, and Latin America are supplied by maize, and maize also serves as a principal fodder crop for livestock across the globe [25]. The United States, China, and Brazil are the top three maize-producing countries in the world, accounting for approximately 563 of the 717 million metric tons/year [26]. In addition, maize currently has the largest production and demand gap.

Given the crucial role of *CRF* genes in regulating plant growth, development, and stress resistance, it is imperative to conduct a comprehensive analysis of the *CRF* gene family in maize and identify genes associated with abiotic stress resistance. In this research, based on maize genome data, we systematically identified members of the CRF family and conducted bioinformatic analysis. Our examination included the chromosomal distribution, gene duplication events, evolutionary relationships, gene structures, motif compositions, promoter cis-regulatory elements, and expression characteristics of all *ZmCRF* genes. Furthermore, qRT-PCR experiments were carried out to validate changes in the expression levels of *ZmCRF* members during various abiotic stresses. Importantly, we generated overexpressing plants and loss-of-function mutants of *ZmCRF9*, respectively. Overexpression of *ZmCRF9* led to enhanced cold and salt tolerance. In contrast, decreased cold and salt tolerance were attained after the application of knockout mutants of *ZmCRF9*. Ultimately, we also analyzed the putative interaction proteins of ZmCRF proteins by illustrating the protein–protein interaction network. Collectively, these findings generated a comprehensive overview of the maize *CRF* gene family and provided the candidate genes for breeding abiotic stress-tolerant maize varieties.

## 2. Results

### 2.1. Identification of CRF Genes in Maize and Sequence Analysis

To determine *CRF* gene members within the maize genome, we initiated a BLASTP search using the amino acid sequences of *Arabidopsis* CRFs (AtCRF1-AtCRF12). Subsequently, SMART (http://smart.embl-heidelberg.de/, accessed on 10 March 2024) was employed to confirm the presence of both CRF and AP2 domains in the candidate *CRF* genes [27]. This comprehensive approach led to the identification of 12 novel maize *CRF* genes, similar to the count in *Arabidopsis* (12). Following this identification, we performed the characterization of these genes, including their genomic locations, open reading frame (ORF) lengths, amino acid numbers (AA), grand averages of hydropathy (GRAVY), iso-electric points (PI), and molecular weights (MW) (Table S1). The ORF lengths ranged from 714 bp (ZmCRF1) to 1152 bp (ZmCRF8), corresponding to full-length proteins spanning 238 to 384 amino acid residues. Among these, ZmCRF8 exhibited the highest molecular weight (42.47 kDa), whereas ZmCRF1 displayed the lowest molecular weight (25.62 kDa). Most maize CRF proteins have a PI below 7, with the lowest being 4.41 (ZmCRF11). Only four members surpass a PI of 7, with the highest recorded at 11.43 (ZmCRF1). Additionally, the GRAVY values for all examined maize CRFs were below zero, indicating that all 12 ZmCRF proteins are hydrophilic (Table S1).

Furthermore, based on amino acid sequences, we conducted a multiple alignment to re-evaluate the members of the maize *CRF* family, followed by a comparison of the detailed sequence information with maize and *Arabidopsis* CRFs. As depicted in Figure S1, a conserved CRF domain at the N-terminal region and an AP2/ERF domain at the center were identified in all represented proteins, aligning with the characteristic features of CRF proteins. With the exception of ZmCRF1, ZmCRF2, ZmCRF7, and ZmCRF8, the remaining ZmCRFs all contain a P site (SP(T/S)SVL motif) at the C-terminal region [9,28]. In *Arabidopsis*, the P site is only present in half of the AtCRFs (AtCRF1-AtCRF6).

### 2.2. Chromosomal Localization and Syntenic Analysis

**Figure 1:**
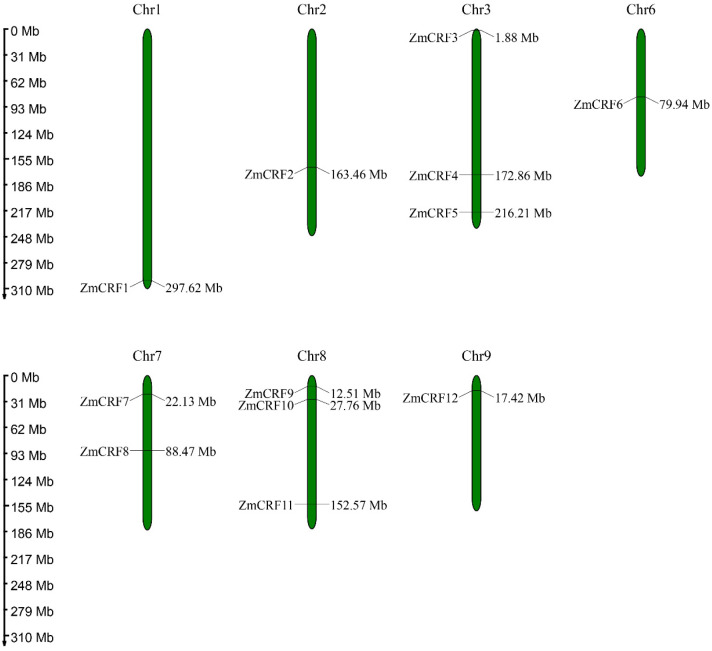
Chromosomal locations of maize CRFs. Twelve ZmCRF genes were found across chromosomes 1, 2, 3, 6, 7, 8, and 9. The corresponding chromosome numbers are denoted at the uppermost section of each chromosome.

To assess the chromosomal localization of 12 *ZmCRFs* within the maize genome, we employed the MapChart program to map them onto the seven maize chromosomes. As shown in Figure 1, chromosome 3 contains three *ZmCRFs* (*ZmCRF3*, *ZmCRF4*, and *ZmCRF5*), while chromosome 8 also comprises three genes (*ZmCRF9*, *ZmCRF10*, and *ZmCRF11*). Additionally, *ZmCRF7* and *ZmCRF8* are situated on chromosome 7, with chromosomes 1, 2, 6, and 9 each featuring one *ZmCRF*.

**Figure 2:**
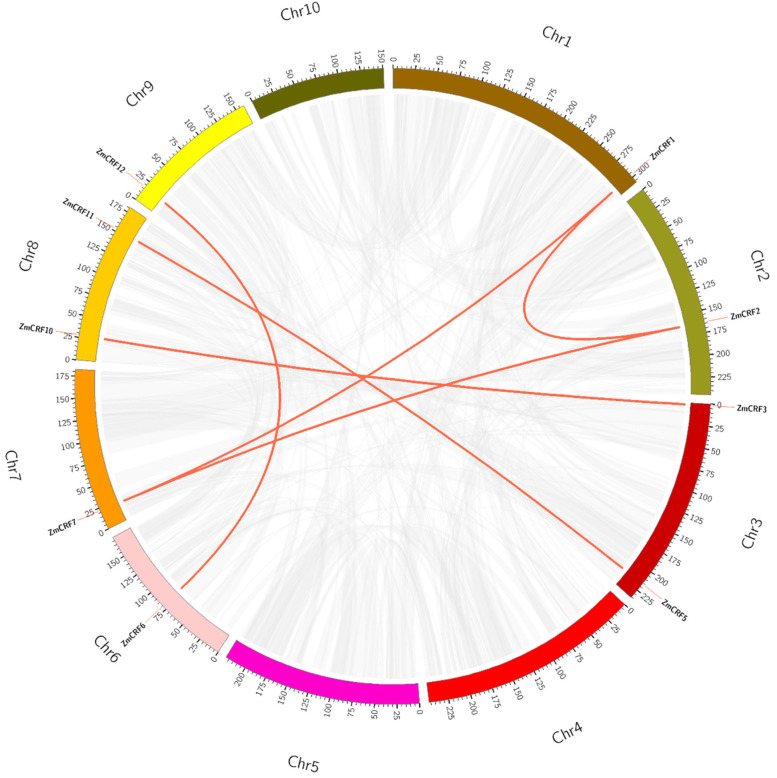
The Circos diagram of the ZmCRF gene family. The red lines connect segmentally duplicated genes. The gray lines depict gene collinearity regions in the maize genome. The color bars signify individual maize chromosomes. Scale bars on chromosomes provide a visual reference for chromosomal lengths in megabases (Mb).

**Figure 3:**
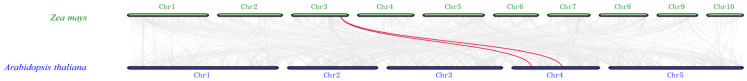
Synteny assessment of CRF genes between maize and Arabidopsis. The collinear blocks within the genomes of both species are indicated by background gray lines, while the syntenic CRF gene pairs are labeled with red lines.

Given the pivotal role of duplication events in gene family expansion during evolution [29,30], we conducted a syntenic analysis within the maize genome. Based on sequence identity and query coverage, six gene pairs (*ZmCRF1/ZmCRF2*, *ZmCRF1/ZmCRF7*, *ZmCRF2/ZmCRF7*, *ZmCRF3/ZmCRF10*, *ZmCRF5/ZmCRF11*, and *ZmCRF6/ZmCRF12*) were identified as segmentally duplicated, distributed across chromosomes 1, 2, 3, 6, 7, 8, and 9. In contrast, *ZmCRF4*, *ZmCRF8*, and *ZmCRF9* did not undergo duplication events (Figure 2). Notably, none of the *ZmCRF* genes in the maize genome were associated with tandem duplication. These findings strongly indicate that segmental duplication events can regulate the expansion of ZmCRFs compared with tandem repeat events. To further evaluate the evolutionary forces in shaping *ZmCRF* genes, we calculated Ka (nonsynonymous substitution rate) and Ks (synonymous substitution rate) for the duplicated gene pairs (Table S2). The Ka/Ks values of the six gene pairs ranged from 0.13 to 0.44, all less than 1, suggesting that these *ZmCRF* genes are evolving under strong purifying selection (Ka/Ks < 1) [31]. Additionally, comparing the genome-wide data of maize *CRFs* with those of *Arabidopsis* identified two orthologous gene pairs, as illustrated in Figure 3.

### 2.3. Phylogenetic Classification of ZmCRF Genes

**Figure 4:**
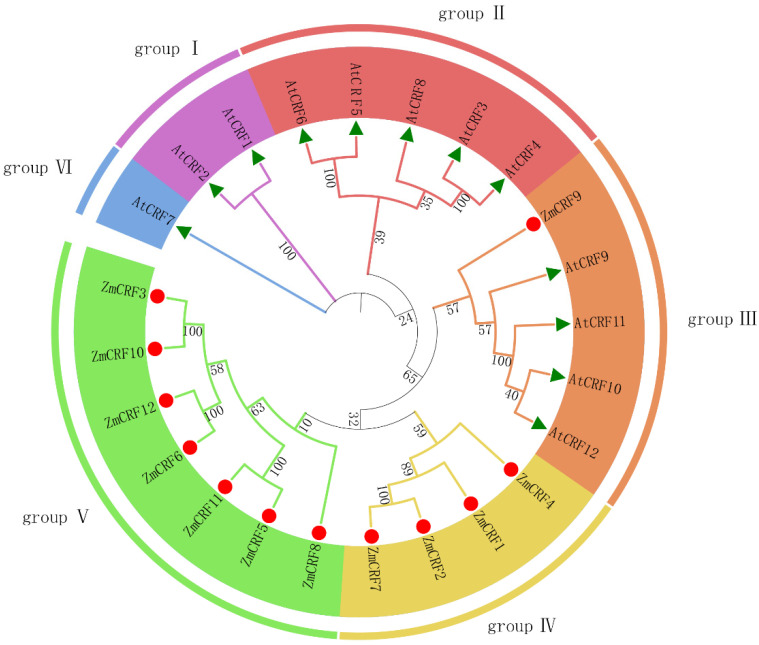
Phylogenetic analysis of CRF proteins in maize and Arabidopsis. The phylogenetic tree, constructed with 1000 bootstrap replicates, was visually enhanced by categorizing it into distinct subfamilies. Each subfamily, denoted by colors, was further labeled as I–VI, representing the six identified subfamilies.

Based on phylogenetic analysis, CRFs have been reported to be classified into different subgroups across various plants, including *Arabidopsis* [8,9], tomato [21], Chinese cabbage [22], *Brassica napus* [16], and soybean [23]. To investigate the phylogenetic relationships among maize CRFs, we generated a phylogenetic tree based on the protein sequences following the Neighbor-Joining (NJ) method. As illustrated in Figure 4, all CRF proteins from maize and *Arabidopsis* were categorized into six subgroups (I to VI). Interestingly, the 12 *AtCRFs* and *ZmCRF9* are distributed across four subgroups (I to VI); among them, group VI only contains one *CRF* member (*AtCRF7*). Meanwhile, all *ZmCRFs*, with the exception of *ZmCRF9*, belong to group IV and group V. Furthermore, when combining the duplication analysis with phylogenetic classification, it appears that duplicated gene pairs exhibit closer evolutionary relationships.

### 2.4. Conserved Motifs and Gene Structures of the ZmCRFs

**Figure 5:**
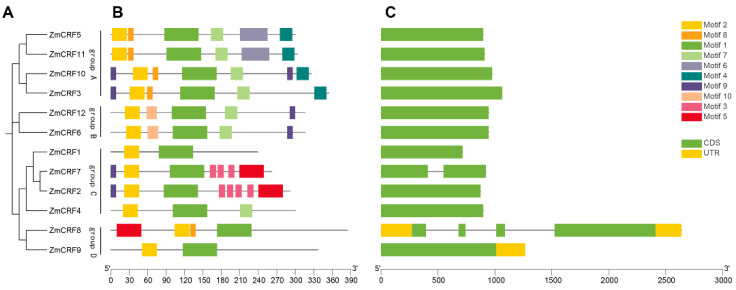
Phylogenetic clustering, conserved motifs, and gene structure of maize CRFs. (**A**) The Neighbor-Joining (NJ) phylogenetic tree of 12 maize CRF proteins. (**B**) Conserved motif distribution map of ZmCRFs. Diverse colored boxes depict the 10 predicted motifs. (**C**) Gene structure of maize CRFs. Green boxes represent the coding region (CDS), while yellow boxes represent the untranslated region (UTR).

From the maize reference genome database (www.maizegdb.org, accessed on 12 March 2024), we obtained the protein sequences of all maize CRFs to independently establish an NJ tree and to analyze the conserved motifs using MEME online software (http://meme-suite.org/meme, accessed on 5 March 2024). As a consequence, we identified ten conserved motifs (Motif 1–10), as shown in Figure S2. Based on phylogenetic analysis and the distribution of motifs, ZmCRFs could be divided into four clusters (Figure 5A). Generally, evolutionally close CRFs exhibit similar structures in terms of motif composition. As illustrated in Figure 5B, among the ten motifs, motif 2 is present in all 12 *ZmCRF* genes, representing the CRF domain. Additionally, motif 1, which represents the AP2/ERF domain, is also distributed in all ZmCRFs. Furthermore, motif 7 at the C-terminal region is absent in members from group C and group D, except for ZmCRF4, aligning with the multiple alignment data mentioned earlier. The remaining motifs mainly occur in ZmCRFs in a group-dependent fashion. For instance, motif 4 is specifically detected in ZmCRFs of group A. Interestingly, ZmCRF2 and ZmCRF7, which exhibit similar structures, are distinct from other members of group C. Notably, the C-terminal region significantly varies among the four groups, contributing to the evolutionary divergence and functional differences of maize CRF proteins.

Gene structure is also responsible for the evolutionary process of gene families [32]. To further determine the diversity of *ZmCRFs*, we assessed the structure of each maize *CRF* gene based on the GSDS database (http://gsds.gao-lab.org/, accessed on 15 March 2024). As shown in Figure 5C, most *CRF* gene members from the same group generally display similar gene structures. For example, four members of group A are composed of just one exon. UTR regions are only found in *ZmCRF8* and *ZmCRF9*, two members of group D. Interestingly, the gene structure of *ZmCRF8* is the most complex, which contains UTR regions, four exons, and three introns.

### 2.5. Cis-Element Analysis in the Promoter Regions of Maize CRF Genes

**Figure 6:**
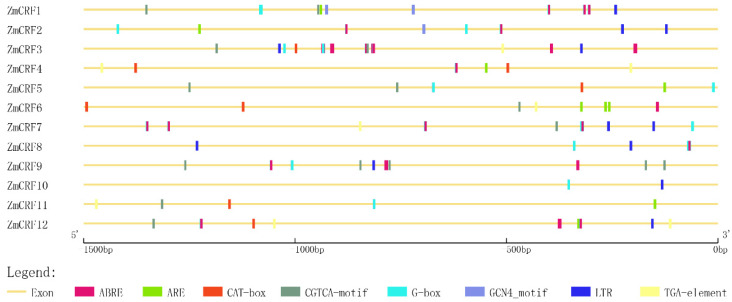
Cis-elements analysis in ZmCRFs promoter regions. Distinct colored boxes at the bottom represent diverse cis-elements.

The promoter region is associated with the expression divergence of a gene under various conditions [33]. To explore the types of cis-acting elements in the promoters of all *ZmCRFs*, we retrieved 1500 bp sequences upstream of the start codon (ATG) for each *ZmCRF* and analyzed them for the presence of cis-regulatory elements using PlantCARE. In total, 21 types of cis-regulatory elements associated with tissue-specific expression, abiotic stress, light response, hormone response, and TF binding were identified in the promoter regions of these *ZmCRF* genes (Figure 6 and Table S3). Upon examining these *cis*-acting elements, we observed that ABA-responsive elements, light-responsive elements, low-temperature-responsive elements, and MeJA-responsive elements were the most common (Figure 6 and Table S3). This strongly suggests that *ZmCRF* genes may play vital roles in hormone signal transduction and responses to multiple abiotic stresses. Additionally, it is noteworthy that the MYB-binding site (CAACTG motif) is also common, indicating that the MYB transcription factor can regulate the expression of the *ZmCRF* gene (Table S3). Interestingly, we also found that some *ZmCRF* promoters contain *cis*-regulatory elements involved in meristem and endosperm expression (Figure 6), highlighting the crucial functions of the *ZmCRF* gene family in meristem and endosperm growth and development.

### 2.6. Expression Profiles of Maize CRFs in Diverse Tissues and Developmental Stages

**Figure 7:**
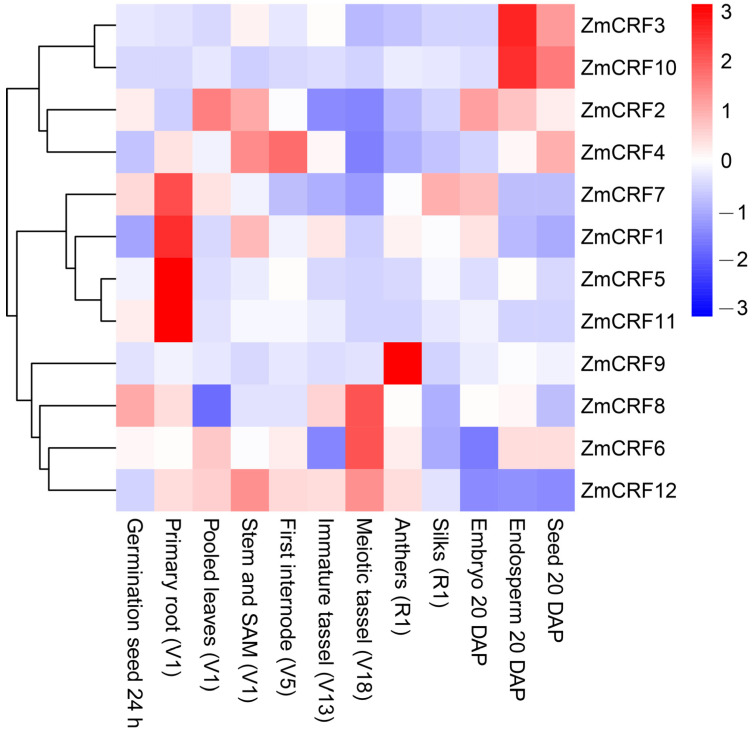
Heatmap of the expression levels of ZmCRFs across diverse tissues or developmental stages. The heatmap illustrates Log2-normalized RPKM values, reflecting gene expression levels.

To elucidate the underlying functions of *ZmCRF*s in maize growth and development, the expression patterns of *ZmCRF*s according to diverse tissues and developmental stages were obtained from the transcriptome data (Maize eFP Browser, http://bar.utoronto.ca/efp_maize/cgi-bin/efpWeb.cgi, accessed on 20 March 2024) [34]. The expression data covered 12 various tissues such as germinating seed 24 h, stem and SAM (V1), primary root (V1), pooled leaves (V1), first internode (V5), immature tassel (V13), meiotic tassel (V18), anthers (R1), silks (R1), embryo 20 DAP, endosperm 20 DAP, and seed 20 DAP. A hierarchical clustering heatmap was established according to these data (Figure 7).

The expression data revealed that all *ZmCRF* genes had significant differential expression in different tissues and stages. Interestingly, some *ZmCRFs* exhibited ubiquitous expression with the highest expression level in a specific tissue. For instance, *ZmCRF3* and *ZmCRF10*, two duplicated *CRFs* from group A, displayed remarkably high expression in endosperm 20 DAP and shared similar expression profiles across diverse tissues and stages. This suggests their potential functional redundancy and key roles in maize endosperm development. In addition, *ZmCRF1*, *ZmCRF5*, *ZmCRF7*, and *ZmCRF11* exhibited high expression in the primary root (V1), while *ZmCRF9* showed the highest expression in anthers (R1). This indicates that *ZmCRF1*, *ZmCRF5*, *ZmCRF7*, and *ZmCRF11* function in primary root growth and development, and *ZmCRF9* acts as a key player in maize anther development. However, further in-depth studies are still required to elucidate the underlying molecular mechanisms.

### 2.7. Expression Profiles of ZmCRFs under Multiple Abiotic Stresses

**Figure 8:**
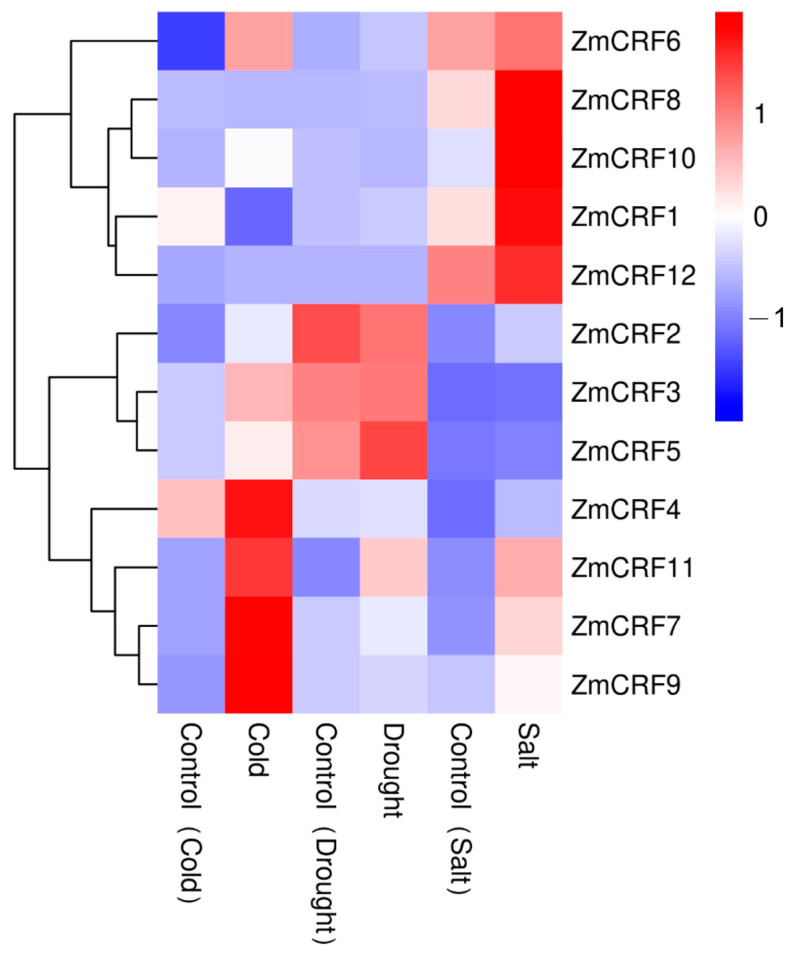
Heatmap of the expression profiles of ZmCRFs under abiotic stresses. The heatmap illustrates Log2-normalized RPKM values, reflecting gene expression levels.

There is increasing evidence illustrating the vital functions of plant CRF proteins in responding to multiple abiotic stresses, including cold, drought, salt, and oxidative stresses [13,14,15,17,18,19,20]. Detailed information from the *cis*-element analysis strongly suggests that maize CRFs might participate in the response to various stress signals. To assess the potential functions of *ZmCRF*s in abiotic stresses, a series of transcriptome sequencing data were retrieved from the maize genome database. Subsequently, a hierarchical clustering tree of *ZmCRF* genes was generated. As shown in Figure 8, under cold stress, the majority of *ZmCRFs* were significantly induced, while two members, *ZmCRF1* and *ZmCRF8*, were downregulated. Similarly, nearly all maize *CRFs*, with the exception of *ZmCRF6*, were upregulated by salt stress. These data strongly suggest that maize *CRFs* play pivotal roles in response to salt and cold stresses. Intriguingly, when plants encounter drought stress, only *ZmCRF11* was slightly upregulated, and the transcript abundances of the remaining members showed almost no change. Hence, the findings imply that *ZmCRFs* might not play a role in governing responses to drought stress.

**Figure 9:**
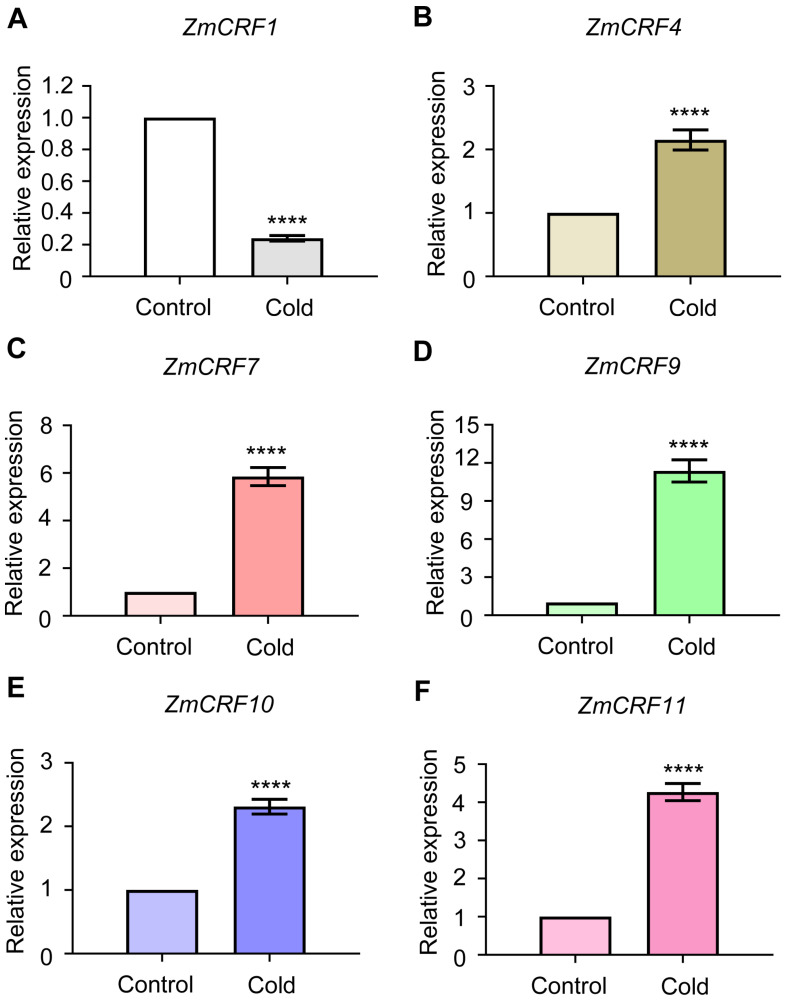
The qRT-PCR analysis of six selected ZmCRF genes in response to cold treatment. Expression in control was set to 1.00. Mean ± SD. **** represent *p* < 0.0001 vs. control (Student’s *t*-test).

**Figure 10:**
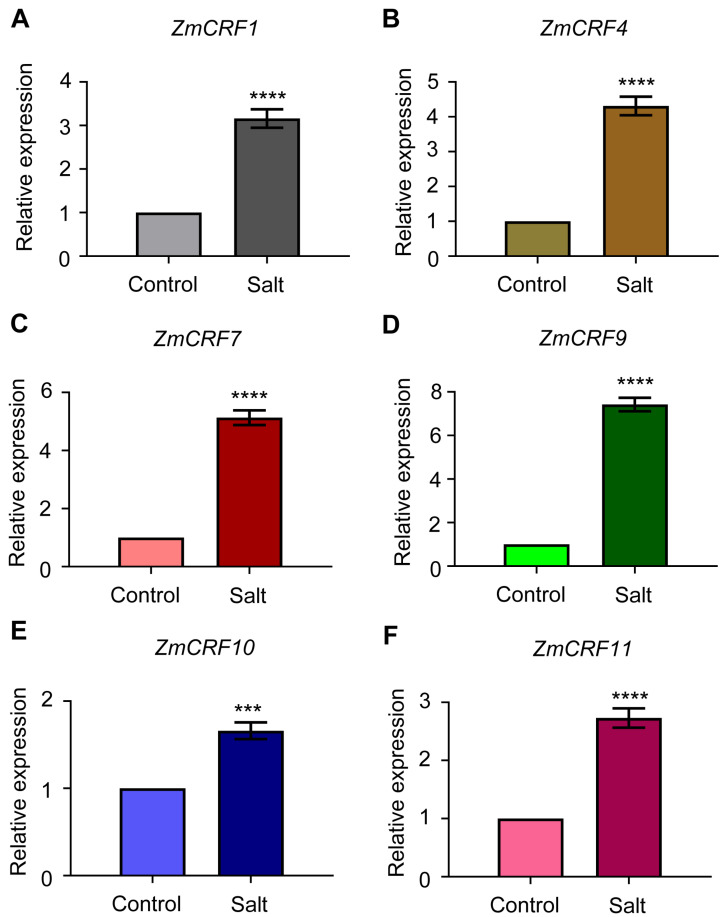
The qRT-PCR analysis of six selected ZmCRF genes in response to NaCl treatment. Expression in control was set to 1.00. Mean ± SD. ***, and **** represent *p* < 0.001, and *p* < 0.0001 vs. control, respectively (Student’s *t*-test).

Furthermore, qRT-PCR analysis was conducted to verify the microarray data. Six genes (*ZmCRF1*, *ZmCRF4*, *ZmCRF7*, *ZmCRF9*, *ZmCRF10*, and *ZmCRF11*) were selected to determine expression changes under different abiotic stresses. As shown in Figure 9A–F, consistent with microarray data, five of six detected genes (*ZmCRF4*, *ZmCRF7*, *ZmCRF9*, *ZmCRF10*, and *ZmCRF11*) are significantly induced by cold stress. On the contrary, the expression level of *ZmCRF1* is obviously downregulated after exposure to cold stress. Under salinity conditions, the transcript abundances of all detected *ZmCRFs* are dramatically upregulated, which is also in line with the above-mentioned microarray data (Figure 10A–F). As expected, only *ZmCRF11* exhibited slight upregulation in response to drought stress (Figure S3A–F). Collectively, these qRT-PCR results further confirm the crucial functions of *ZmCRFs* in regulating cold and salt stresses but not drought stress and also implicate that different *ZmCRFs* may govern molecular mechanisms under salt and cold stresses.

### 2.8. ZmCRF9 Functions as a Positive Regulator of Cold and Salt Tolerance in Maize

**Figure 11:**
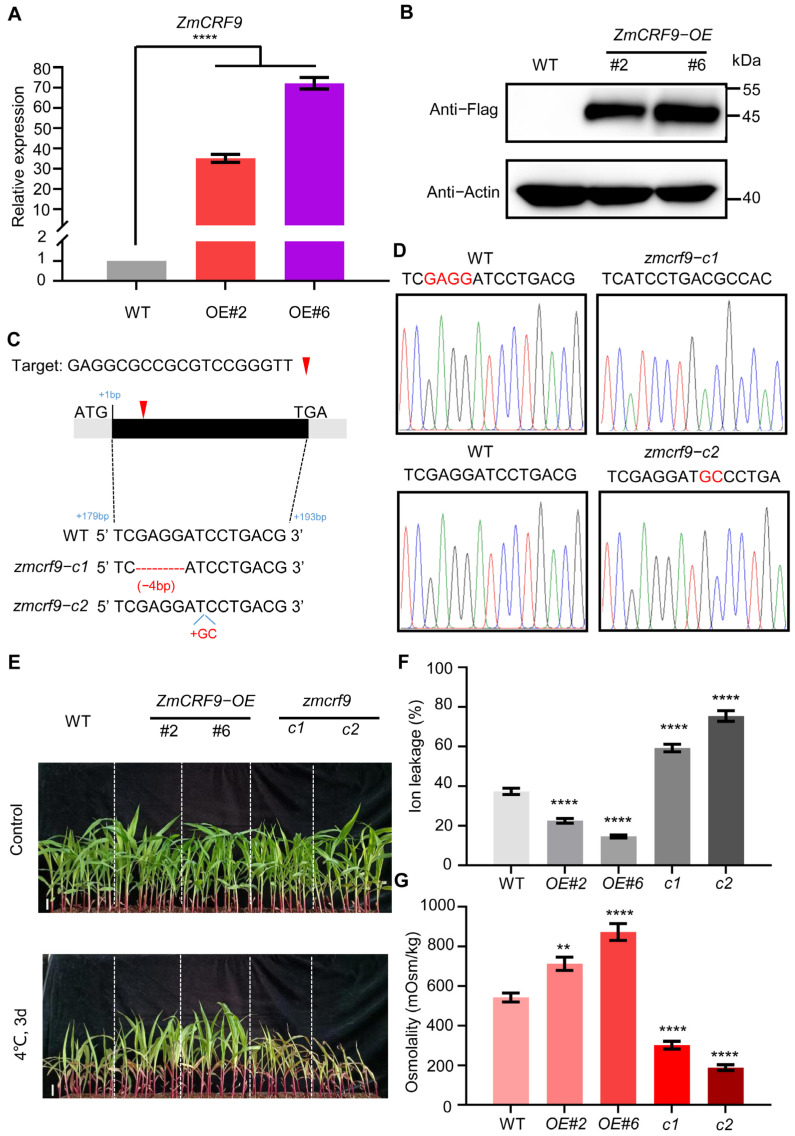
ZmCRF9 positively regulates cold tolerance in maize. (**A**) The transcript abundance of ZmCRF9 in WT and ZmCRF9-OE seedlings. Expression in WT was set to 1.00. Data shown are means ± SD of three biological replicates. **** represents *p* < 0.0001 vs. control (Student’s *t*-test). (**B**) The protein abundance of ZmCRF9 in ZmCRF9-OE transgenic seedlings (#2, #6). Anti-Flag antibody was utilized to detect ZmCRF9. Actin served as a control. (**C**) Schematic diagrams of zmcrf9-crispr mutants generated by CRISPR/Cas9-mediated genome editing. Different colored lines indicate four different bases. (**D**) The zmcrf9-c1 and zmcrf9-c2 mutations were identified by Sanger sequencing compared with WT. (**E**–**G**) Cold phenotypes (**E**), ion leakage (**F**), and osmolarity (**G**) of WT, ZmCRF9-OE, and zmcrf9-crispr plants. Fourteen-day-old seedlings grown at 25 °C were exposed to 4 °C for 3 days, followed by recovered at 25 °C for 1 day. Scale bars = 5 cm. In (**F**,**G**), data shown are means ± SD (n = 3 seedlings per replicate) of three biological replicates. **, and **** represent *p* < 0.01, and *p* < 0.0001 vs. control, respectively (Student’s *t*-test).

To further address the biological function of ZmCRFs in response to multiple abiotic stresses, ZmCRF9 was selected due to its highest induction upon cold and salt stresses. Overexpression lines and loss-of-function mutants of *ZmCRF9* were generated. Post-examining transcript level and protein abundance, we selected two independent overexpression plants (*ZmCRF9-OE#2* and *ZmCRF9-OE#6*) for detailed study (Figure 11A,B). Through employing the CRISPR/Cas9 genome editing system [35,36], we also obtained two loss-of-function mutants of *ZmCRF9*, named as *zmcrf9-c1* and *zmcrf9-c2*. *zmcrf9-c1* harboring a 4 bp deletion (from 181 to 184 bp downstream of ATG), while *zmcrf9-c2* carried a 2 bp insertion (186 bp downstream of ATG), resulting in frameshifts in the ORF and premature termination of translation (Figure 11C,D).

Following incubation at 4 °C for 3 d, *ZmCRF9-OE* plants showed a cold-tolerant phenotype, while *zmcrf9-crispr* plants exhibited a cold-sensitive phenotype in comparison to WT plants (Figure 11E). Ion leakage and osmolarity serve as indicative markers of cold tolerance [37,38]; consistent with corresponding phenotypes, subsequent to cold stress, compared with those of WT plants, reduced ion leakage and greater osmolarity were accompanied in *ZmCRF9-OE* plants, whereas *zmcrf9-crispr* plants showed increased ion leakage and weaker osmolarity (Figure 11F,G). These outcomes strongly advocated for ZmCRF9 as a positive regulator of cold tolerance in maize.

**Figure 12:**
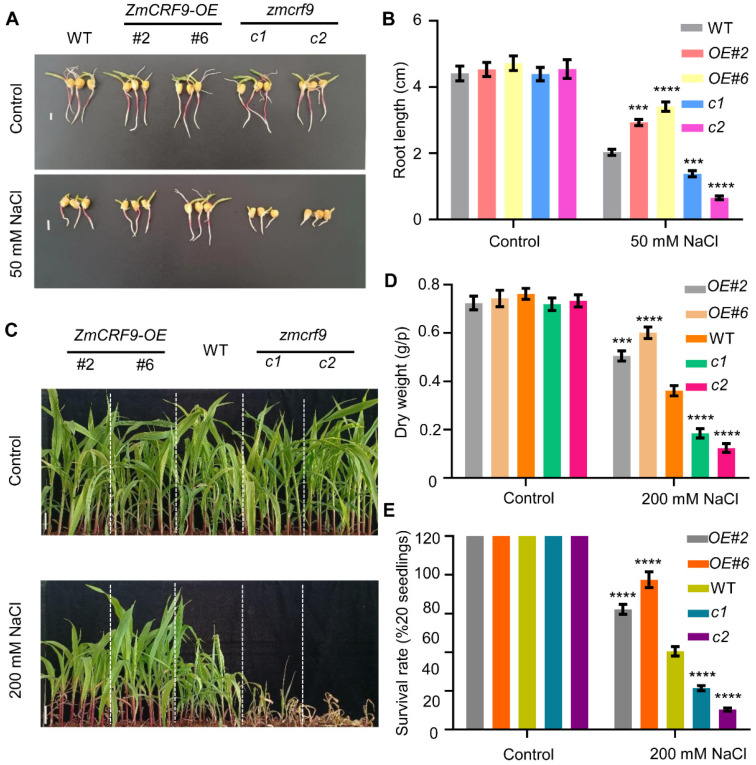
ZmCRF9 confers salt tolerance in maize. (**A**) The root growth phenotype of WT, ZmCRF9-OE, and zmcrf9-crispr maize seeds under salt stress. Scale bars = 1 cm. (**B**) Statistical analysis of root length in (**A**). Data are means of three biological replicates ± SD (n = 90). *** and **** represent *p* < 0.001 and *p* < 0.0001 vs. control, respectively (Student’s *t*-test). (**C**) Salt stress phenotype of WT, ZmCRF9-OE, and zmcrf9-crispr seedlings. Scale bars = 5 cm. (**D**,**E**) Dry weight and survival rate of WT, ZmCRF9-OE, and zmcrf9-crispr seedlings under normal and salt stress conditions. Data are means of three biological replicates ± SD (n = 60 for (**D**), and n = 12 for (**E**)). ***, and **** indicate significant difference to the corresponding controls with *p* < 0.001 and *p* < 0.0001, respectively (Student’s *t*-test).

Furthermore, we also extended our inquiry to assess the involvement of ZmCRF9 in the response to salt stress. Seeds of WT, *ZmCRF9-OE*, and *zmcrf9-crispr* were soaked in a solution containing 50 mM NaCl until the root length was measured. As shown in Figure 12A,B, without NaCl treatment, similar root length was observed among WT, *ZmCRF9-OE*, and *zmcrf9-crispr* seeds. In contrast, after salt treatment, *ZmCRF9-OE* seeds displayed significantly longer root lengths, whereas root-growth inhibition was obviously enhanced in *zmcrf9-crispr* seeds compared with the WT. Subsequently, two-week-old seedlings of WT, *ZmCRF9-OE*, and *zmcrf9-crispr* were treated with water (control) or 200 mM NaCl. A similar phenotype was observed among WT, *ZmCRF9-OE*, and *zmcrf9-crispr* under normal growth conditions. In contrast, compared with WT, *ZmCRF9-OE* plants grew better and *zmcrf9-crispr* plants suffered more damage when treated with 200 mM NaCl for 12 d, respectively (Figure 12C). Additionally, after salt treatment, compared with those of WT plants, dry weight and survival rate were significantly higher in *ZmCRF9-OE* plants and dramatically lower in *zmcrf9-crispr* plants, respectively (Figure 12D,E). These data strongly indicated that ZmCRF9 functions as a positive regulator of salt tolerance in maize.

To assess drought tolerance, 12-day-old seedlings of WT, *ZmCRF9-OE*, and *zmcrf9-crispr* were conducted with drought stress without watering for 8 days, followed by 3 days of rewatering. Intriguingly, in terms of growth performance, survival rate, relative water content, MDA content, and electrolyte leakage, no overt distinctions were discerned among WT, *ZmCRF9-OE*, and *zmcrf9-crispr* plants under conditions of normal, drought stress, and rehydration (Figure S4). These results suggested that ZmCRF9 is not involved in drought stress response in maize. In summation, based on the aforementioned findings, ZmCRF9 emerges as a positive regulator specifically implicated in cold and salt tolerance, while remaining unrelated to drought tolerance.

### 2.9. Interaction Network Analysis of ZmCRF Proteins

**Figure 13:**
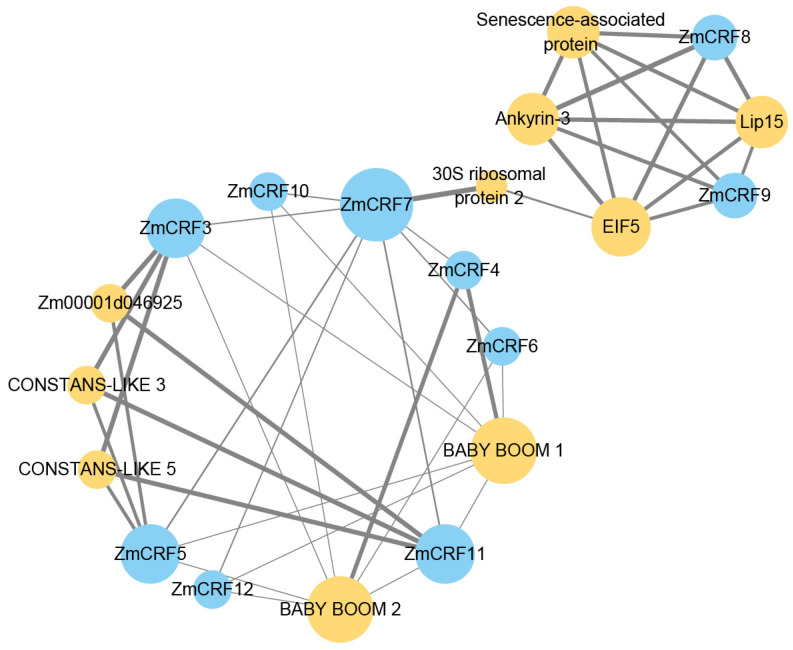
Predicted protein–protein interaction networks (PPI) using the STRING tool. Within the PPI network, every node encapsulates all proteins derived from the corresponding single gene. The size of each node reflects the degree of interaction, while the thickness of the edges signifies the strength of protein–protein interactions. Nodes representing ZmCRFs are depicted in blue, while proteins interacting with ZmCRFs are highlighted in yellow.

To dissect the biological functions and regulatory networks of *ZmCRF* genes, we constructed a protein–protein interaction network based on *Arabidopsis* orthologous proteins. The network comprises 19 nodes (genes) and 40 edges (regulatory relationships). As illustrated in Figure 13, several proteins associated with diverse molecular and genetic processes exhibit robust interactions with ZmCRF proteins. For instance, CONSTANS-LIKE 3 is linked to the control of photoperiod response and flowering time, regulation of circadian rhythms, and light signal transduction [39], while low temperature-induced protein 15 (Lip15) is responsible for plant cold-stress response [40]. Taken together, these findings further underscore the multifaceted roles of *ZmCRF* genes, not only in maize growth and development but also in the response to multiple abiotic stresses.

## 3. Discussion

The pivotal roles of CRF proteins in plant growth, development, and response to various stresses are well-established. Despite substantial progress in exploring *CRF* gene families across diverse species via whole genome analyses, a comprehensive study of this family in maize has been lacking. Herein, we performed a genome-wide analysis and identified 12 *CRFs* in maize, which has a genome size of 2106 Mb [41]. Interestingly, previous studies revealed that *Arabidopsis*, tomato, Chinese cabbage, *Brassica napus*, and soybean have 12, 11, 21, 44, and 26 *CRF* genes, respectively, with genome sizes of 157, 950, 384, 840, and 1115 Mb [42,43,44,45,46], respectively. These findings suggest that the number of *CRF* genes may not be directly correlated with genome size, although the underlying mechanisms warrant further investigation.

Conserved motifs are amino acid sequences crucial for various biological functions, encompassing nuclear localization, protein–protein interaction, and transcriptional activation [47]. Notably, while motif 1 (the CRF domain) and motif 2 (the AP2/ERF domain) are present in all ZmCRFs, significant motif divergence at the C-terminal is observed in four subfamilies of ZmCRFs. This aligns with the proposed notion that the C-terminal region of CRFs is clade-specific and may be implicated in lineage-specific functions [11]. Overall, ZmCRFs, with their distinct conserved motifs, likely exhibit evolutionary and functional diversity, a hypothesis that necessitates further experimental validation.

The organizational structure of a gene is believed to be intricately linked to its function and the gene family’s evolutionary trajectory [32]. Exons carry the essential information necessary for cellular protein synthesis [48], while introns act as protective elements, shielding coding proteins from the occurrence of randomly generated deleterious mutations [48]. Interestingly, 10 out of the 12 *ZmCRF* genes consist of a single exon, representing 83.33% of all family members. Similar scenarios have been observed in other species, with ratios of 84.09%, 90.48%, and 92.31% reported in *Brassica napus* [16], Chinese cabbage [22], and soybean [23], respectively. We hypothesize that this phenomenon may stem from the evolutionary conservation inherent in the *CRF* gene family across diverse species. However, further investigations are necessary to substantiate this hypothesis.

*Cis*-acting regulatory elements of the putative promoters are responsible for tissue-specific expression and multiple stress resistance [49]. Utilizing the PlantCARE online software (http://bioinformatics.psb.ugent.be/webtools/plantcare/html/, accessed on 8 March 2024), we anticipated the presence of hormone-response elements, abiotic stress-related elements, and tissue-specific expression-related elements in *CRF* promoter regions. Remarkably, analogous patterns of elements have been documented in Chinese cabbage and soybean, indicating a ubiquitous association between *CRF* genes and hormonal and developmental regulatory mechanisms, as well as stress responses in plants. However, more extensive biochemical and genetic evidence is required to comprehensively elucidate the underlying mechanisms.

To date, transcriptome data remain the primary data source for unraveling the bioinformatic features of various gene families [50]. Herein, we assessed the transcription levels across diverse tissues and treatments using transcriptome data from the maize genome database (Maize eFP Browser). It is noteworthy that certain *ZmCRF* genes display ubiquitous expression patterns, reaching their highest expression levels in specific tissues. For instance, *ZmCRF9* exhibited peak expression in anthers (R1), suggesting that different ZmCRF members may serve specific functions in distinct tissues or developmental stages during growth. Moreover, RNA-seq data and qRT-PCR analysis demonstrated that the majority of *ZmCRF* genes are induced by salt and cold stresses, underscoring their potential roles in responding to these environmental challenges. This aligns with recent studies in model species such as *Arabidopsis*, implicating CRFs in diverse abiotic stress responses such as salt, oxidative, and cold stresses. Surprisingly, the transcript abundances of nearly all ZmCRF members remained largely unchanged in response to drought conditions. Given that salinity negatively impacts plant growth through ionic and osmotic stress, along with secondary stresses induced by reactive oxygen species, and drought induces osmotic stress [51,52], our speculation is that ZmCRFs may specifically respond to ionic or oxidative stress triggered by salinity in maize. Nevertheless, a more profound understanding of the detailed molecular mechanisms requires further investigation.

Notably, transcriptome data clearly suggested that there was a strict separation among the paralogues responding to salt (*ZmCRF1*, *ZmCRF8*, *ZmCRF10*, and *ZmCRF12*) and the paralogues responding to cold (*ZmCRF4*, *ZmCRF7*, *ZmCRF9*, and *ZmCRF11*). Herein, we searched for evidence to explain this differentiation regarding promoter analysis, gene structure, and the synteny. In terms of promoter analysis, interestingly, 5, 2, 1, and 5 ABRE elements (cis-acting element involved in the abscisic acid responsiveness), which may be closely related to salt response, were distributed on the promoter regions of *ZmCRF1*, *ZmCRF8*, *ZmCRF10*, and *ZmCRF12*, respectively. Surprisingly, most ABRE elements (10) were found on the promoter region of *ZmCRF3*. In addition, with the exception of *ZmCRF4*, *ZmCRF5*, *ZmCRF6*, *ZmCRF9*, and *ZmCRF11*, the LTR elements (cis-acting element involved in low-temperature responsiveness) were present on the promoter regions of the remaining *ZmCRFs*, and two LTR elements were detected on the promoter region of *ZmCRF7*. As a consequence, these findings indicated that the data from promoter analysis may not provide significant evidence associated with expression patterns of *ZmCRF* genes. As for gene structure, intriguingly, no similar structure was observed among the paralogues responding to salt or the paralogues responding to cold. For example, the most complex structure was described in ZmCRF8, which showed significant differences with ZmCRF1, ZmCRF10, and ZmCRF12. Furthermore, a syntenic analysis also pointed out no orthologous gene pair existed in the paralogues responding to salt or the paralogues responding to cold. Hence, further investigations are required to comprehensively elucidate the underlying mechanisms.

In summary, the comprehensive analysis and deep investigation of ZmCRF9 conducted here aimed to systematically explore the *CRF* gene in the major crop plant, maize. A precise understanding of regulatory systems and molecular mechanisms will establish a theoretical foundation for future genetic improvements.

## 4. Material and Methods

### 4.1. Identification and Bioinformatics Analysis of the ZmCRF Genes

To identify candidate *CRF* genes in maize, the protein sequences of 12 known Arabidopsis CRFs were retrieved from The Arabidopsis Information Resource (TAIR, http://www.arabidopsis.org/index.jsp, accessed on 19 February 2024). Subsequently, through employing default parameters and a significant e^−3^ value, a BLASTP search was conducted in the maize genome sequences (B73 RefGen_v4), which were obtained from the Ensembl Plants database (https://plants.ensembl.org/index.html, accessed on 19 February 2024). Next, on the basis of Pfam (http://pfam.xfam.org/, accessed on 19 February 2024) and NCBI CDD (https://www.ncbi.nlm.nih.gov/cdd, accessed on 19 February 2024) databases, we further confirmed the presence of CRF and AP2 domains of candidate ZmCRF protein sequences. In addition, to investigate the protein properties of ZmCRFs, the ExPASY website (https://www.expasy.org/, accessed on 20 February 2024) was employed to calculate various parameters, including the number of amino acid residues, grand average of hydropathicity (GRAVY), isoelectric points (pI), and molecular weights (MW).

### 4.2. Characterization of Chromosomal Locations

Based on the maize reference genome information (B73 RefGen_v4), we successfully mapped all maize *CRF* genes to their respective locations. Subsequently, the MapChart software (v2.0) was utilized to construct the chromosomal localization map of 12 *ZmCRF* genes [53].

### 4.3. ZmCRF Gene Duplication and Synteny Analyses

To investigate the inter-chromosomal relationships of *ZmCRFs* genes, the segmental and tandem duplication events were obtained from Multiple Collinearity Scan toolkit X version software (MCScanX) with the default settings within the maize genome (B73 RefGen_v4) [54]. The obtained data were visually presented using the Advanced Circos module in TBtools [55]. At least two genes located on the same chromosome, one by one, are considered as tandem duplications, while segmental duplications are identified with a feature of being on different chromosomes or distributing on the same chromosome without being close together [56]. Gene family members often evolve from an ancestral gene. To explore synteny between *ZmCRFs* and orthologous *AtCRF* genes from *Arabidopsis*, synteny analyses were conducted using the Dual Synteny Plotter module in TBtools. To further assess the selection pressure on *ZmCRF* genes, key parameters such as non-synonymous substitution rates (Ka), synonymous substitution rates (Ks), and Ka/Ks ratio were calculated. Notably, Ka/Ks ratios greater than, equal to, and less than 1 were considered to represent positive, neutral, and negative selection, respectively. This analysis, providing insights into the evolutionary dynamics and selection pressures, was carried out using TBtools software (v2.0).

### 4.4. Comparative Sequence Alignment and Phylogenetic Assessment

To assess the evolutionary relationships, the full-length protein sequences of CRFs from *Arabidopsis* and maize (12 ZmCRFs and 12 AtCRFs) were subjected to multiple sequence alignment using MUSCLE software (v3.6) [57]. Subsequently, a phylogenetic tree was constructed using the MEGA_X_10.1.7 program, employing the neighbor-joining NJ method with default parameters and 1000 bootstrap replicates with the following parameters: poisson correction, complete deletion, and 1000 bootstrap replicates [58], and the constructed phylogenetic tree was visualized with the online EvolView (https://evolgenius.info/, accessed on 26 February 2024).

### 4.5. Conserved Motifs and Gene Structure Analysis

Utilizing the protein sequences of maize CRF proteins, conserved motif analysis was conducted with MEME (http://meme-suite.org/meme, accessed on 5 March 2024) [59], with the following parameters: a maximum of 10 motifs and motif width ranging from 6 to 50 amino acids. The gene structures, comprising untranslated region (UTR) composition, introns, and coding sequences (exons) of the 12 *ZmCRF* genes, were elucidated using the GSDS2.0 (http://gsds.gao-lab.org/, accessed on 7 March 2024) program [60]. All the above results were visualized using TBtools software.

### 4.6. Cis-Acting Regulatory Element Analysis of ZmCRFs

For each *ZmCRF* gene family member, the putative promoter region was defined as the 1.5 kb upstream genome sequence. Identification of cis-regulatory elements was conducted by analyzing the 1.5 kb promoter sequences of all 12 *ZmCRF* genes using the PlantCARE (http://bioinformatics.psb.ugent.be/webtools/plantcare/html/, accessed on 8 March 2024) [61]. The resulting graph was constructed using TBtools software.

### 4.7. Expression Profiles of ZmCRFs in Maize

Transcriptome data for 12 *ZmCRF* genes across diverse tissues (germinating seed 24 h, stem and SAM (V1), primary root (V1), pooled leaves (V1), first internode (V5), immature tassel (V13), meiotic tassel (V18), anthers (R1), silks (R1), embryo 20 DAP, endosperm 20 DAP, and seed 20 DAP) and abiotic stresses (cold, salt, and drought) were retrieved from Maize eFP Browser (http://bar.utoronto.ca/efp_maize/cgi-bin/efpWeb.cgi, accessed on 10 March 2024) and referenced in prior studies [62]. Then, acquired data were converted into logarithmic values to draw and decorate hierarchical clustering heatmaps using TBtools software.

### 4.8. Plant Materials and Growth Conditions

The OE lines and CRISPR-Cas9 mutants were crafted by Wimi Biotechnology (Jiangsu) Co., Ltd., situated in Changzhou, China. The corresponding maize seeds were planted and cultivated in a meticulously controlled growth chamber (Percival, Des Moines, IA, USA). The environment within the chamber was upheld at a temperature of 25 °C, a 16 h photoperiod featuring 400 µmol m^−2^ s^−1^ light intensity, and a relative humidity of 70%.

We performed the cold treatment upon a reported program [63]. A 4 °C handle was conducted on 14-day-old maize seedlings in a cold chamber, followed by recovery at 25 °C for 1 day before photography. We conducted the ion leakage assay according to a reported method [64]. We obtained the leaves from chilling-treated maize seedlings, and then, corresponding leaves were immersed in 10 mL deionized water of 15 mL centrifuges. After vacuuming for 0.5 h, the conductance of the water was measured and named as S0. Before being detected as S1, the solution was shaken for 60 min. Subsequently, the samples were boiled for 0.5 h, followed by cooling to room temperature. The ion concentration was measured and named as S2. The value of (S1 − S0)/(S2 − S0) was calculated as ion leakage. Osmolality was detected with an OsmoTECH PRO Multi-Sample MicroOsmometer [64]. The plant extract was prepared from the leaves of 14-day-old maize plants (following treatment at 4 °C for 24 h) and directly passed through a 1 mL medical syringe. Osmolality was measured utilizing twenty microliters of samples.

The salt treatment was performed as reported previously [65]. Briefly, 14-day-old maize seedlings were treated with a 200 mM NaCl solution. Twelve days later, comprehensive photographic documentation of the plants was undertaken. Following this, the dry weight of the aerial components of the plants was quantified post-exposure to 200 mM NaCl for a duration of 15 days. In addition, the survival rate of the plants was intricately calculated after enduring a 35-day treatment with 200 mM NaCl.

The drought treatment protocol was implemented as outlined in [66]. The 12-day-old maize seedlings underwent an 8-day water deprivation period, followed by a subsequent 3-day rehydration phase. Phenotypic observations were documented, and the survival rate was computed. Relative water content was performed as reported previously [67]. Leaf samples were collected for malondialdehyde (MDA) content analysis, involving homogenization in 10% trichloroacetic acid with 0.65% 2-thiobarbituric acid (TBA), followed by heating at 100 °C for 15 min, as in [68]. The percentage of electrolyte leakage was assessed following the protocol outlined in [69].

Seedlings at the three-leaf stage were exposed to cold (4 °C), salinity (200 mM NaCl solution), and drought (10% PEG 4000) stress conditions for the designated duration.

### 4.9. RNA Isolation and qRT-PCR

Total RNA extraction from maize tissues was performed following meticulous protocols using the FastPure^®^-Universal-Plant-Total-RNA-Isolation-Kit (Vazyme, Nanjing, China). Subsequently, 1 µg of the extracted total RNA underwent reverse transcription using the HiScript^®^-III-RT-SuperMix-for-qPCR(+gDNA-wiper)-kit provided by Vazyme. qRT-PCR was performed using the ChamQ-Universal-SYBR-qPCR-Master-Mix on a Stratagene-Mx3000P-real-time-system-cycler (Agilent, Santa Clara, CA, USA). The actin1 gene (GRMZM2G126010) served as the reference control. Each run included three technical replicates, and three independent biological experiments were conducted. Detailed information on the qRT-PCR primers can be found in Table S4.

### 4.10. Plant Transformation

The coding sequence of *ZmCRF9* was amplified and subsequently endowed with a Flag tag at their C termini. Following this, the resulting DNA fragments were integrated downstream of the ubiquitin promoter. The requisite target site, strategically positioned within the inaugural exon of ZmCRF9 to facilitate the creation of CRISPR/Cas9 knockout lines, was sourced from CRISPR-P (http://crispr.hzau.edu.cn/CRISPR2/, accessed on 1 June 2022). The acquisition of transgenic plants was executed through the meticulous process of Agrobacterium-mediated transformation. For a more nuanced exploration, the subsequent analyses were conducted exclusively on the homozygous T4 overexpression (OE) lines. Additionally, in the ensuing experiments, the homozygous CRISPR-Cas9 mutant lines-void of the Cas9 transgene served as pivotal participants. It is imperative to underscore that this facet of the investigation was adroitly executed by Wimi Biotechnology. Comprehensive details pertaining to the primers can be referenced in Table S4.

### 4.11. Statistical Analysis

The examination of datasets comprising two distinct groups entailed a thorough implementation of the Student’s *t*-test, where the designation “ns” indicates the absence of a statistically significant difference relative to the corresponding controls. The symbols “**”, “***”, and “****” are employed to denote a significant difference from the respective controls, with *p*-values falling beneath the thresholds of 0.01, 0.001, and 0.0001, respectively. A form of mean ± standard deviation (SD) was used to present the data.

## 5. Conclusions

In conclusion, this research successfully identified and characterized 12 *ZmCRF* genes within the maize genome, offering a comprehensive analysis of their family traits. Evolutionary and synteny analyses highlighted the significant role of segmental duplications in shaping the maize genome. Conserved motif and gene structure analyses unveiled commonalities within different subgroups. The presence of diverse cis-regulatory elements in *ZmCRF* gene promoters, including those associated with plant growth, hormones, and abiotic stress responses, was systematically explored. Expression profile heatmaps underscored tissue-specific patterns and responses to multiple abiotic stresses in the maize *CRF* gene family. Validation through qRT-PCR experiments on six *ZmCRF* genes further affirmed their potential roles in abiotic stress responses. In addition, deep investigation demonstrated the positive role of ZmCRF9 in regulating cold and salt stresses. Ultimately, an investigation into the protein–protein interaction network shed light on potential interactors of ZmCRF proteins. Collectively, this systematic overview of the *ZmCRF* gene family and deep investigation of ZmCRF9 lay the groundwork for further investigation on the maize CRFs, aiming to enhance maize productivity and stress resilience. A thorough exploration of *ZmCRF* genes remains crucial for advancing our understanding and improving agricultural outcomes.

## Data Availability

Dataset available on request from the authors.

## Supplementary Materials

The supporting information can be downloaded at: https://www.mdpi.com/article/10.3390/ijms25147650/s1.
