# Supplementary material for: Genome-Wide Investigation of the CRF Gene Family in Maize and Functional Analysis of ZmCRF9 in Response to Multiple Abiotic Stresses
Source: Int J Mol Sci. 2024 Jul 12;25(14):7650. doi: 10.3390/ijms25147650 (PMC11276700; doi:10.3390/ijms25147650)
Supplement: Supplementary file 1 [file ijms-25-07650-s001.zip › Supplementary data-CRF.pdf]

Genome-wide investigation of the *CRF* gene family in maize and functional analysis of ZmCRF9 in response to multiple abiotic stresses

Zhenwei Yan[1], Jing Hou[2], Bingying Leng[1], Guoqi Yao[1], Changle Ma[3], Yue Sun[4], Fajun Zhang[1,*], Chunhua Mu[1], Xia Liu[1,*]

[1]Maize Research Institute, Shandong Academy of Agricultural Sciences, Jinan 250100, China; yanzwplant@sina.com (Z.Y.); lengbingying305@126.com (B.L.); yaoguoqi@saas.ac.cn (G.Y.); muchunhua@saas.ac.cn (C.M.)

[2]School of Agriculture, Ludong University, Yantai 264001, China; houjing@m.ldu.edu.cn (J.H.)

[3]College of Life Sciences, Shandong Normal University, Jinan 250300, China; machangle@sdnu.edu.cn (C.M.)

[4]College of Agronomy, Qingdao Agricultural University, Qingdao 266109, China; sunyue3070601@163.com (Y.S.)

*Correspondences: Fajun Zhang (zhangfajun@saas.ac.cn) and Xia Liu (Liuxiamaize@163.com, Dr. Liu is responsible for the distributions of the material associated with this article)

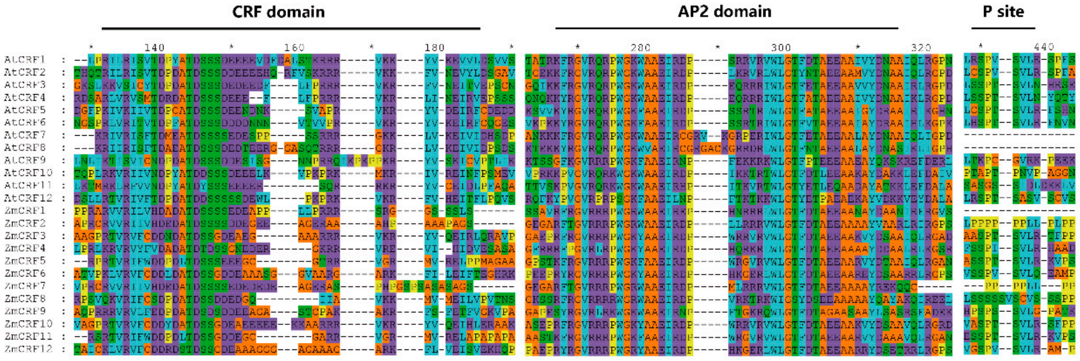

Figure S1. Alignment of CRF proteins in maize and *Arabidopsis*. The CRF domain, AP2 domain, and P site are indicated by black lines above the alignment.

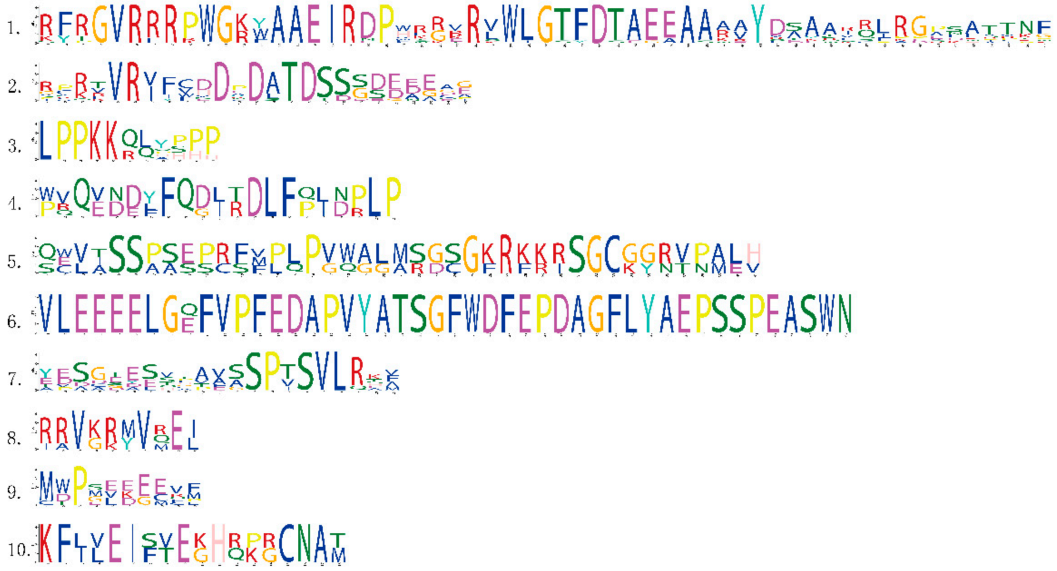

Figure S2. Ten conserved motifs of *ZmCRF* genes.

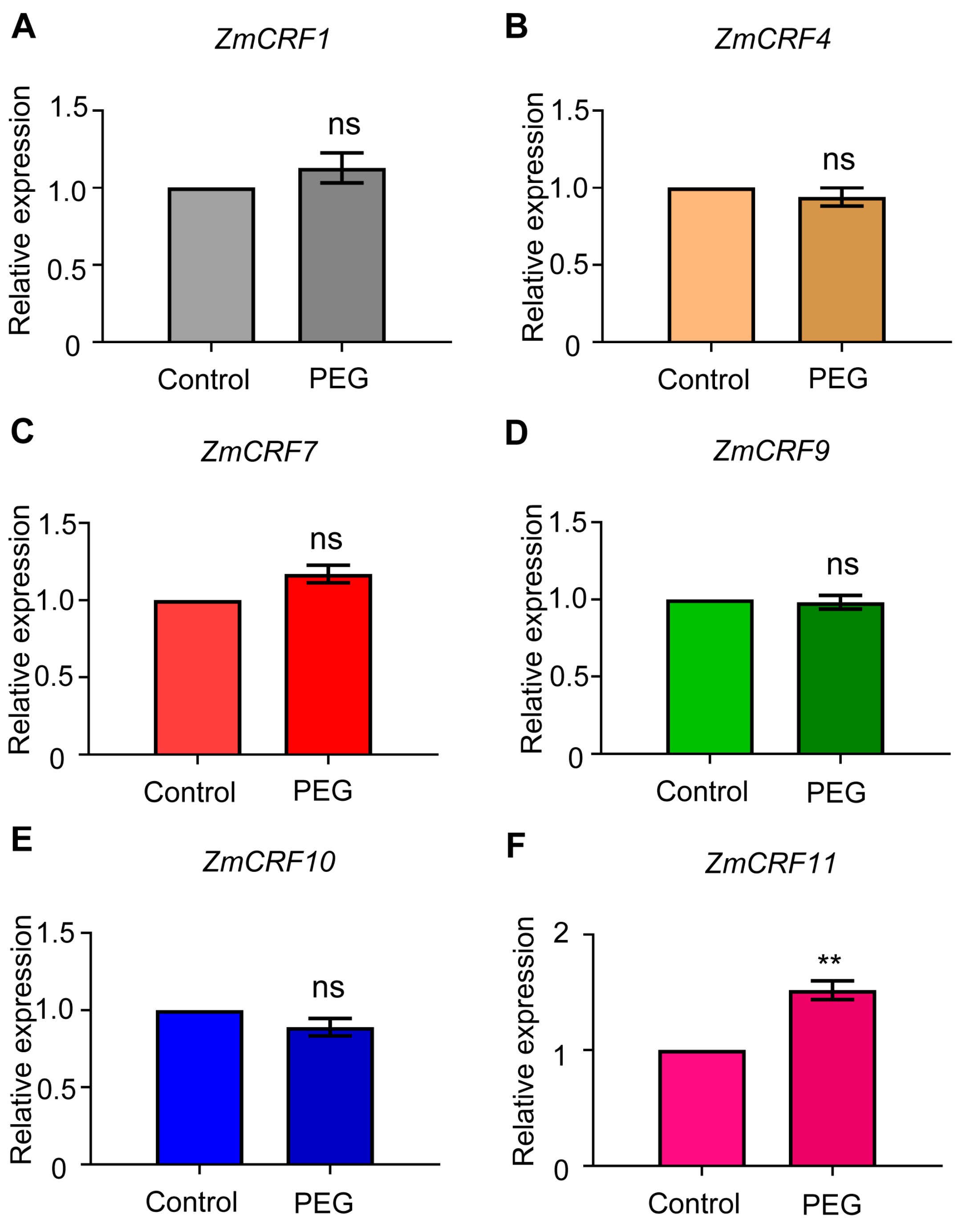

Figure S3. The qRT-PCR analysis of six selected *ZmCRF* genes in response to PEG treatment. Expression in control was set to 1.00. Mean±SD. Ns and ** represent not significant and P<0.01 vs. control, respectively (student's *t*-test).

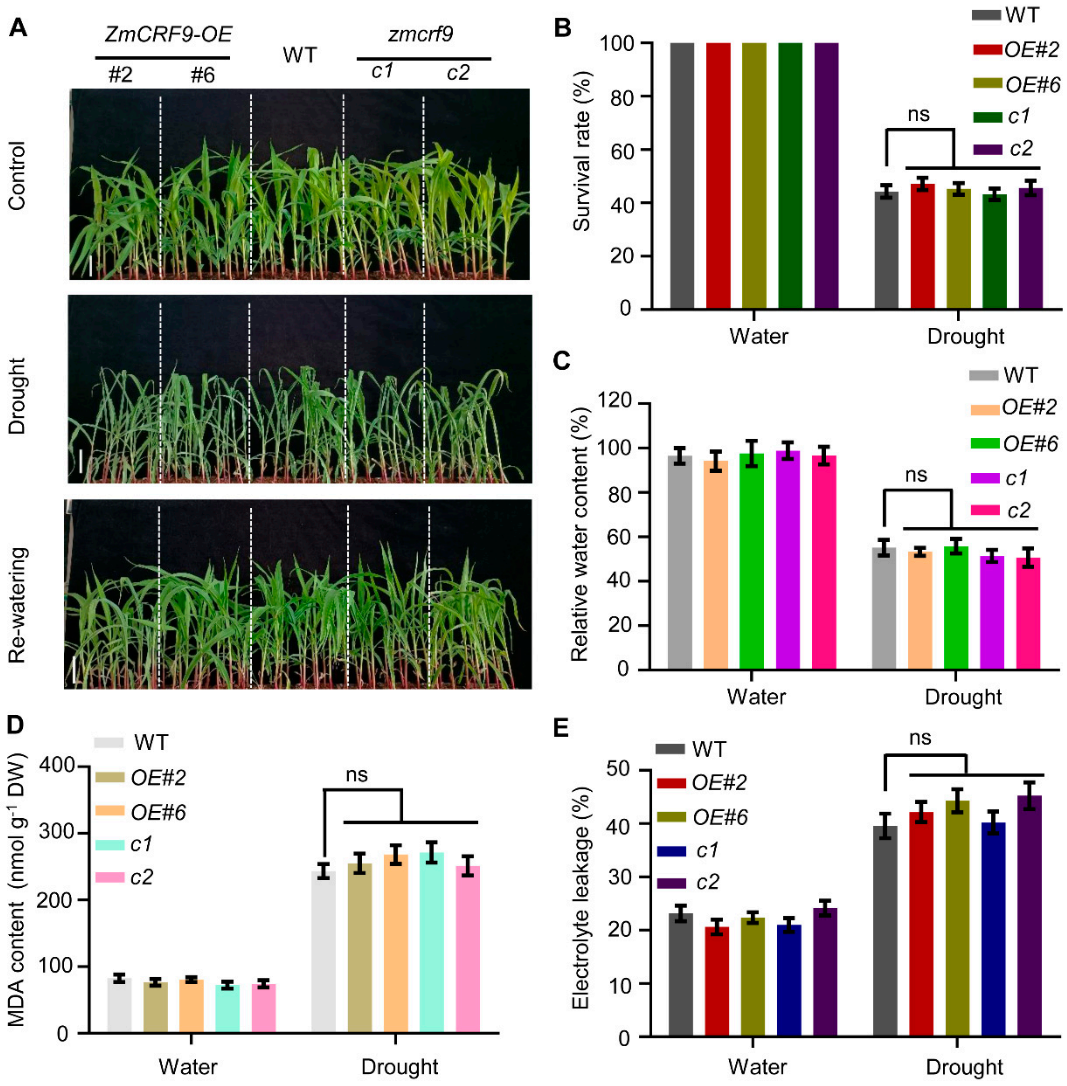

Figure S4. ZmCRF9 is not involved in drought stress response in maize. (A) Drought tolerance phenotypes of WT, *ZmCRF9-OE*, and *zmcrf9-crispr* plants. Twelve-day-old seedlings were subjected to drought stress by withholding water for 8 days, after which they were re-watered for 3 d (Re-watering). Scale bars = 5 cm. (B) Statistical analysis of survival rates after drought stress as shown in (A). At least 30 seedlings of each line per replicate were used for survival rate analysis. Data shown are means ± SD of three biological replicates. Ns represents not significant vs. control (student's *t*-test). (C) Relative water content (RWC) in WT, *ZmCRF9-OE*, and *zmcrf9-crispr* plants under well-watered and drought conditions. Data shown are means ± SD of three biological replicates. Ns represents not significant vs. control (student's *t*-test). (D, E) Malondialdehyde (MDA) content (D) and percentage leakage of electrolyte (E) of

WT, *ZmCRF9-OE*, and *zmcrf9-crispr* plants under well-watered and drought conditions. DW, dry weight. The values are presented as means ± SD of three biological replicates. Ns represents not significant vs. control (student's *t*-test).
